# ASO Author Reflections: Extracapsular Spread in Melanoma Lymphadenopathy: Prognostic Implications, Classification, and Management

**DOI:** 10.1245/s10434-020-09197-9

**Published:** 2020-10-20

**Authors:** Michelle Lo, Howard Peach, Marc Moncrieff

**Affiliations:** 1 Plastic and Reconstructive Surgery Department, Norfolk and Norwich University Hospital, Norwich, UK; 2 Plastic Surgery, Leeds Teaching Hospitals NHS Trust, Leeds, UK; 3 Norwich Medical School, Faculty of Medicine and Health Sciences, University of East Anglia, Norwich, UK

## Past

The current American Joint Committee on Cancer (AJCC) classification system for stage III cutaneous melanoma is heterogeneous, with 5-year melanoma-specific survival rates ranging from 88% for stage IIIA disease to 24% for stage IIID disease. Currently, the stage III classification not only incorporates data from the N stage, but also qualifies T data from the primary tumour such as Breslow thickness and ulceration status. This takes into account the fact that the risk of distant spread and subsequent death from melanoma for the stage III group is dependent on the risk of hematogenous spread from a deeply invading primary tumour, in addition to the disease burden of the metastatic nodes. Extracapsular spread (ECS), a well-known risk factor for locoregional recurrence in melanoma patients with macrometastatic disease, is associated with a poor prognosis.^1^–^3^ Although ECS is a biomarker for poor prognosis, it is not included in the current AJCC classification system.

## Present

This multi-center retrospective cohort study of 515 patients with nodal metastases^4^ comprehensively analyzed the clinical relevance of ECS as a biomarker in nodal disease and demonstrated that ECS is the most important prognostic indicator for both macroscopic and microscopic disease, upstaging patients to the next echelon in a manner comparable with ulceration of the primary. The authors have devised a simplified clinically and surgically relevant classification system for microscopic and macroscopic stage III disease by replacing N stage with ECS status.

## Future

Extracapsular spread was an exclusion criterion for the last two major phase 3 melanoma surgical trials,^5^^,^^6^ and the authors suggest that ECS in sentinel node-positive patients still may be an indication for lymphadenectomy to achieve regional control of disease as well as for adjuvant systemic therapy. In future versions of the AJCC guidelines, ECS should be considered as an independent staging variable pending further validation of its relevance.
